# Recent insights into eukaryotic translation initiation factors 5A1 and 5A2 and their roles in human health and disease

**DOI:** 10.1186/s12935-020-01226-7

**Published:** 2020-04-29

**Authors:** Gao-Qi Wu, Yan-Ming Xu, Andy T. Y. Lau

**Affiliations:** Laboratory of Cancer Biology and Epigenetics, Department of Cell Biology and Genetics, Shantou University Medical College, Shantou, Guangdong 515041 People’s Republic of China

**Keywords:** Eukaryotic translation initiation factor, eIF5A1, eIF5A2, Hypusination, Post-translational modifications, Human cancers, Biomarkers

## Abstract

The eukaryotic translation initiation factor 5A1 (eIF5A1) and its homolog eIF5A2 are the only two human proteins containing the unique post-translational modification–hypusination, which is essential for the function of these two proteins. eIF5A1 was initially identified as a translation initiation factor by promoting the first peptide bond formation of protein during translation; however, recent results suggest that eIF5A1 also functions as a translation elongation factor. It has been shown that eIF5A1 is implicated in certain human diseases, including diabetes, several human cancer types, viral infections and diseases of neural system. Meanwhile, eIF5A2 is overexpressed in many cancers, and plays an important role in the development and progression of cancers. As multiple roles of these two factors were observed among these studies, therefore, it remains unclear whether they act as oncogene or tumor suppressor. In this review, the recent literature of eIF5As and their roles in human diseases, especially in human cancers, will be discussed.

## Background

Humans harbor two proteins of the eukaryotic translation initiation factor 5As, namely eIF5A1 and its homolog eIF5A2, encoded by the genes *EIF5A1* and *EIF5A2*, respectively. Relatively speaking, eIF5A1 is the major eIF5As expressed in the cell and it cDNA sequence was determined in 1989 [1] and *EIF5A1* gene was mapped on chromosome 17p13.1 [2]. *EIF5A2* gene was isolated and sequenced in 2001 and was mapped on chromosome 3q26.2 [3, 4].

eIF5A1 (previously designated as eIF-4D) was first isolated and purified from high salt ribosomal extract of rabbit reticulocyte lysates in 1978 [5]. It can stimulate the reaction of initiator methionyl-tRNA with puromycin when added to an 80S initiation complex, which is a classical assay to simulate the formation of the first peptide bond during protein translation. However, the formation of 80S initiation complex does not require eIF5A1, so eIF5A1 was proposed to exert its function after the formation of the 80S initiation complex, i.e., promoting the formation of the first peptide bond [6]. Recently, functional studies of its yeast homolog also suggest its role in translation elongation and termination [7], especially in the translation of polyproline motifs [8, 9]. In human cells, it has also been reported to promote the translation elongation of specific mRNAs [10]. Although there is also a putative protein encoding gene which is highly homologous to eIF5A1 known as eIF5A1-like (eIF5AL1) in humans, however, so far it has only been validated at transcript level and no research data can be found in the literature.

*EIF5A2* was identified as an oncogene, and in recent years, a growing amount of research has confirmed that *EIF5A2* is involved in cancer development and progression. While homozygous depletion of *EIF5A1* caused an early embryonic lethal phenotype in mice [11], mice with homozygous depletion of *EIF5A2* were viable, fertile, and did not show an obvious difference in body weight or survival time as compared with control mice [12]. These results suggest that *EIF5A2* may be a promising cancer therapeutic target.

Throughout the years, although eIF5A2 is considered more to be related to cancer development and as a potential biomarker, however, we want to emphasize that besides acting as a translation initiation/elongation factor, there is also evidence that eIF5A1 is implicated in certain human diseases, including diabetes, several human cancer types, viral infections, and diseases of neural system.

In the current manuscript, we therefore wish to summarize and give an update on the regulations of expression, post-translational modifications (PTMs), subcellular localization, turnover, and the roles of eIF5As (including both eIF5A1 and eIF5A2) in human diseases, especially in human cancers, in which our review covers all the recent advances of these two factors.

## Dissecting the eIF5As

### Characteristics and general structure of human eIF5A proteins

As because of an additional upstream start codon on *EIF5A1* transcript, there are two isoforms of eIF5A1 protein, eIF5A1 isoform 1 (the canonical one) with 154 residues and eIF5A1 isoform 2 with an additional 30 residues in the N-terminus, and the additional amino acid sequence presents in eIF5A1 isoform 2 is a mitochondrial targeting signal that connects the function of this protein to the mitochondria [13]. Additionally, the first 19 residues of eIF5A1 work as a nuclear localization signal in B16-F10 cells [14]. The minimum domain of the eIF5A1 protein needed for hypusine modification was identified as residues 20–90 [15, 16], and amino acids mutational analyses confirmed that four residues (lysine 47, histidine 51, glycine 52, and lysine 55) are important for hypusine formation [17]. According to the X-ray crystallography data, eIF5A1 protein comprises of two domains with an approximate boundary at residue 83 [18]. The N-terminal domain comprises of six β-strands and a one-turn 3_10_-helix, and contains the hypusine modification site, lysine 50, in the loop connecting β3 and β4, while the C-terminal domain is made up of a three-turn α-helix and five β-strands.

**Fig. 1:**
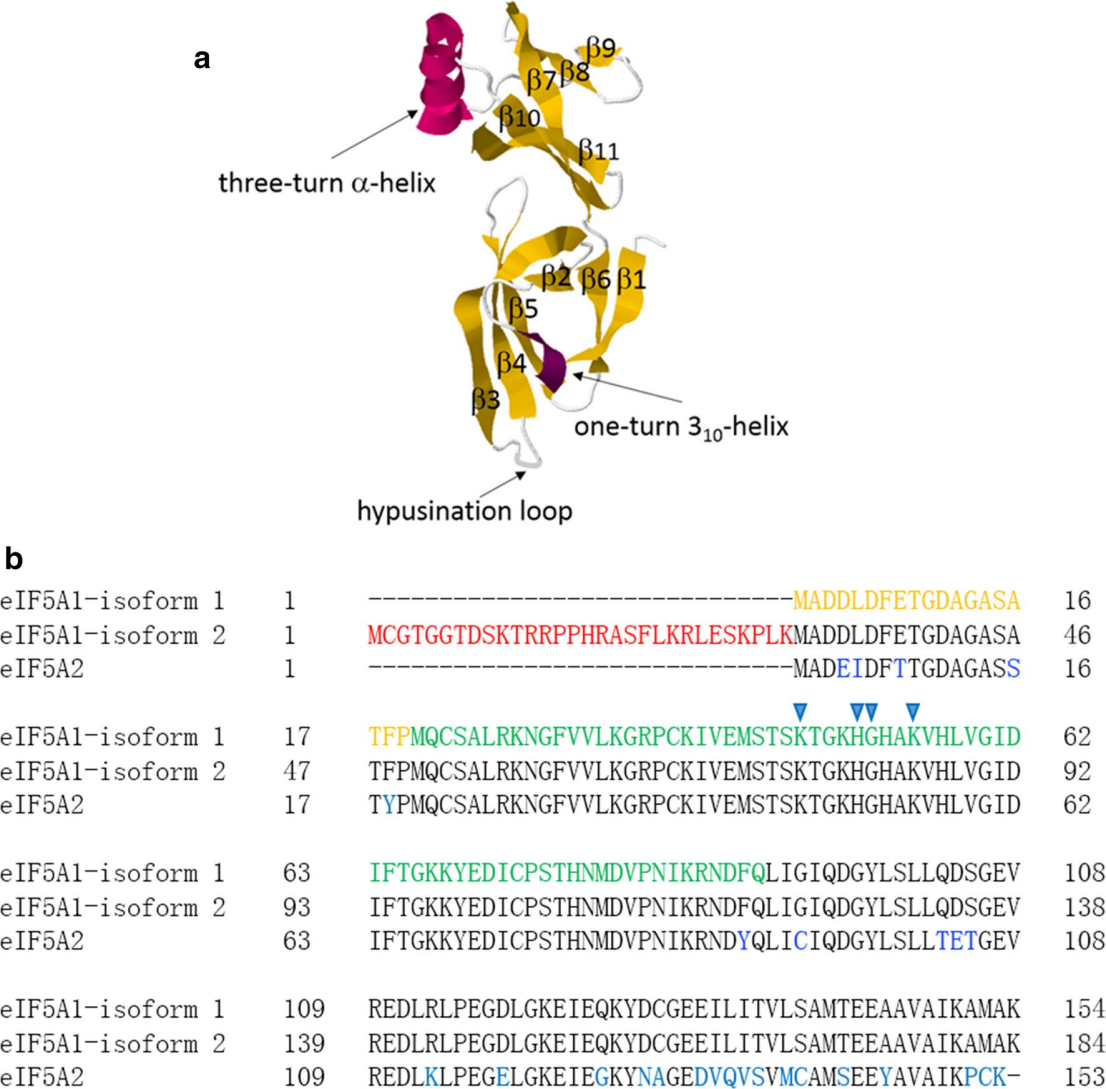
Characteristics and general structure of human eIF5A proteins. **a** Structure of human eIF5A1 (PDB: 3CPF), colors are used to highlight the secondary structures of eIF5A1, eleven β-strands (gold), a one-turn 3_10_-helix (purple), and a three-turn α-helix (purplish red). The hypusine modification site (lysine 50) is located at the loop connecting β3 and β4. **b** alignment of the amino acid sequences of eIF5A1 isoform 1, eIF5A1 isoform 2, and eIF5A2. Red color indicates an additional 30 residues in the N-terminus of non-canonical eIF5A1 isoform 2, blue color for non-matching amino acid residues between eIF5A1 and eIF5A2, orange color for nuclear localization signal for eIF5A1 in B16-F10 cells and green color for the minimum domain of the eIF5A1 protein needed for hypusine modification. Four blue arrowheads indicate lysine 47, histidine 51, glycine 52, and lysine 55, which are important for the hypusine formation

eIF5A2 protein consists of 153 residues, and shares 82% amino acid identity (126 of 153) with eIF5A1. So far, there is no X-ray crystallography data of eIF5A2 protein. However, structural differences between eIF5A1 and eIF5A2 were demonstrated by lack of immunological cross-reactivity between polyclonal antibodies raised against eIF5A1 or eIF5A2, and different kinetic parameters for association with deoxyhypusine synthase (DHS) in vitro [19]. For the sake of simplicity, we summarized and presented the information of this section in Fig. 1.

### Expression regulations of eIF5As

Little is known about the mechanisms by which the expression of *EIF5A1* gene is regulated, but it was documented that KRas 12th/13th codon mutated lung adenocarcinoma has an increased eIF5A1 protein level [20]. What is more, overexpression of KRas G12D in human pancreatic nestin-positive cells induced *EIF5A1* expression at both mRNA and protein levels, while knockdown of activated KRas has an opposite effect [21]. Therefore, these results indicate that the expression of *EIF5A1* can be upregulated via the KRas signaling pathway. On the other hand, the relationship between eIF5A1 protein level and p53 nuclear accumulation was also reported [20]. Additionally, *EIF5A1* gene was identified as a p53 target by protein expression profiling, and a p53-binding motif in the first intron of *EIF5A1* gene was found [22]. Thus, p53 is an upstream regulator of *EIF5A1*. Recently, it was reported that NF-kappaB also plays a role in the expression regulations of *EIF5A1*, which can bind to the promoter region of *EIF5A1* gene, and its binding on *EIF5A1* promoter can be enhanced by cadmium treatment [23]. Non-coding RNAs and epigenetic factors have recently been implicated in regulating *EIF5A1* expression. For instance, miR-434-3p and DNA methylation co-regulate *EIF5A1* in rat skeletal muscle [24]. Another example is long non-coding RNA FOXD1-AS1 which increases the expression level of eIF5A1 protein in a post-transcriptional way [25].

**Fig. 2:**
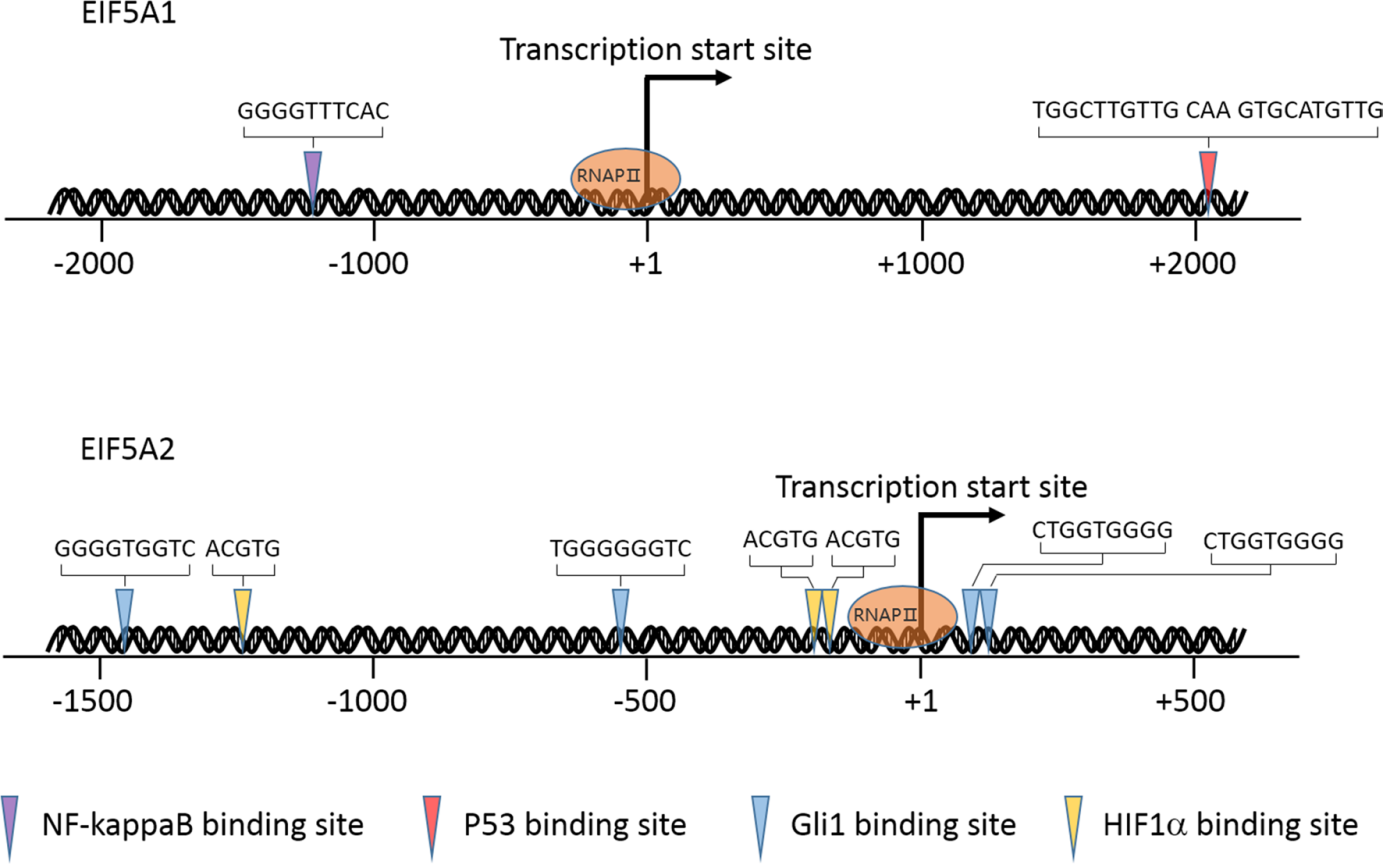
*Cis*-regulatory elements of *EIF5A1* and *EIF5A2* gene. The *EIF5A1* gene contains one NF-kappaB binding site in the promoter region (as demonstrated by ChIP assay) and one putative p53 binding site in the first intron. The promoter region of *EIF5A2* gene contains four Gli1 binding sites and three HIF1α binding sites

Like *EIF5A1* gene, the expression of *EIF5A2* can be upregulated by KRas [21]. Recently, it was reported that *EIF5A2* expression is induced by hypoxia, and was in part via hypoxia-inducible factor 1α (HIF1α), since the promoter region of human *EIF5A2* contains three HIF1α binding sites [26]. Sonic hedgehog (Shh) signaling pathway has a key role in controlling cell growth and differentiation through the activation of transcriptional activator GLI family zinc finger protein 1 (Gli1). Gli1 which binds to the DNA consensus sequence 5′-GACCACCCA-3′ to regulate the transcription of specific genes was also demonstrated to bind to the promoter region of *EIF5A2* gene, and four predicted Gli1 binding sites showing 89% homology with the sequence 5′-GACCACCCA-3′ were demonstrated to mediate the effect of Gli1 on *EIF5A2* expression [27]. For the sake of simplicity, we summarized and presented the information of this section in Fig. 2.

Up to now, several papers reported that miRNAs were implicated in post-transcriptional regulation of *EIF5A2* expression. MiR-30b, miR-125b, miR-203, miR-588, miR-221-3p, and miR-599 were proven to regulate the expression of *EIF5A2* by directly targeting the 3′-UTR of *EIF5A2* mRNA [28–33]. Also, the eIF5A2 protein was regulated by miR-33b, miR-383, and miR-9, but how these miRNAs regulated eIF5A2 protein would need to be further studied [34–36].

### PTMs on human eIF5As

#### Hypusination

Hypusine was first identified in 1971 as a new amino acid, and its occurrence in protein of mammals was demonstrated [37, 38], however, the effort to find the specific protein which contained hypusine was failed until 1983, when translation initiation factor eIF-4D (subsequently named eIF5A1) was proven to be the hypusine-containing protein [39]. Up to now, it is believed that hypusine only occurs in eIF5A proteins, which is formed by a chemical modification of the lysine 50 residue, and two enzymes are responsible for this modification—DHS and deoxyhypusine hydroxylase (DOHH). Firstly, DHS catalyzes the transfer of the butylamine portion derived from the spermidine to the side chain of a lysine residue to form a deoxyhypusine, and subsequently the incoming aminobutyl portion was hydroxylated by DOHH. Hypusination is needed for eIF5A1 function, for instance, unhypusinated eIF5A1 form fails to stimulate the synthesis of methionyl-puromycin [40].

Both eIF5A1 and eIF5A2 are shown to be hypusinated at lysine 50, and it is worth noting that the exogenously produced eIF5A1 and eIF5A2 resulted in increased levels of the unhypusinated form in human cells because of the limited activity of DHS and DOHH [41, 42]. To inhibit the formation of hypusine, N1-guanyl-1,7-diaminoheptane (GC7) and ciclopirox are commonly used as an inhibitor of DHS and DOHH, respectively [43].

#### Acetylation

Both eIF5A1 and eIF5A2 can be acetylated at lysine 47, and their subcellular distributions were affected by this modification [41, 44]. Additionally, it was observed that hypusine residue of eIF5A1 was also acetylated, which results in acetylhypusine, and acetylation of hypusine residue impairs the eIF5A1 activity on methionyl-puromycin synthesis assay [45]. Further results revealed that the cellular acetyltransferase responsible for the acetylation of eIF5A1 lysine 47 was histone acetyltransferase PCAF, while histone deacetylase 6 and NAD-dependent protein deacetylase sirtuin-2 were identified as its major deacetylases [44]. However, hypusine residue was selectively acetylated by spermidine/spermine N(1)-acetyltransferase 1 [45]. Interestingly, exogenous transfection assay confirmed that hypusination inhibits acetylation because of the importance of basic charge at residue 50 for acetylation at lysine 47 [42].

#### Sulfation

Protein tyrosine sulfation, which was initially reported in 1954, was mainly found in transmembrane and secreted proteins, and implicated in multiple biochemical processes experimentally demonstrated in recent years, e.g., the recognition between chemokine receptor and chemokine [46]. It was estimated that sulfated proteins account for 7% of mammalian proteins, however, the subsequent significance of this PTM on proteins under biological and physiological conditions is not fully appreciated and understood [47]. Tyrosine sulfated-eIF5A1 was firstly isolated and identified among secreted proteins of cardiac myocytes treated with hypoxia/reoxygenation, and the 69th residue was identified as sulfated tyrosine. What is more, the authors found that sulfated-eIF5A1 induced apoptosis of cultured cardiac myocytes [48]. They also observed that sulfated-eIF5A1 plays a role in oxidative stress-induced apoptosis, and elevation of sulfated-eIF5A1 concentration was found in the vitreous body from proliferative diabetic retinopathy patients, which may explain the molecular mechanism of oxidative stress-induced retinal injury in diabetic retinopathy [49]. A secretome analysis of human lung cells treated with cadmium chloride showed that cadmium can induce the secretion of eIF5A1 [50], however, it is unclear whether the secreted eIF5A1 was tyrosine sulfated and its link to cadmium-induced oxidative stress would need further exploration.

#### Subcellular localization

Though it was firstly reported that eIF5A1 is mainly localized in the nucleus as a host co-factor required for the function of viral trans-activator protein Rev of the human immunodeficiency virus type 1 (HIV-1) [51], another study showed that eIF5A1 is primarily localized in the cytoplasm in COS-7, NIH3T3, CV-1, and HeLa S3 cells, but is nearly undetectable in the nucleus [52]; however, more recent evidences suggest that eIF5A1 is a shuttling protein, and presents in the nucleus, cytoplasm, and mitochondrion. First, a whole-cell distribution pattern of eIF5A1 in living cells was observed by direct visualization of GFP-tagged eIF5A1, and it can cross the barrier of nuclear pore via passive diffusion [53]. The nuclear export of eIF5A1 is more complex, which requires the help of exportin-1 or exportin-4, and its nuclear export can be blocked by exportin-1 inhibitor leptomycin B or deletion of exportin-4 [54–56]. Also, eIF5A1 was located in the mitochondria; evidence has shown that IGF2 mRNA-binding protein 1 prevents mitochondrial accumulation of eIF5A1 by binding with eIF5A1 in the cytoplasm, which inhibits eIF5A-mediated apoptotic effect [57]. In addition, the dynamic balance of eIF5A1 subcellular distribution also can be affected by PTMs including acetylation and hypusination. It was demonstrated that hypusine modification of eIF5A1 is critical for the interaction with exportin-4 [55, 58], and non-hypusinated eIF5A1 is found in both the cytoplasm and nucleus, while hypusinated eIF5A1 appeared to be excluded from the nucleus [42]. On the other hand, acetylation of eIF5A1 at lysine 47 results in the nuclear accumulation of eIF5A1 [44].

eIF5A2 protein is also a shuttling protein, and like the eIF5A1 protein, knockdown of exportin-4 leads to nuclear accumulation of eIF5A2 protein suggesting that exportin-4 mediates the export of eIF5A2 protein from the nucleus [56]. Similar to eIF5A1 protein, hypusinated eIF5A2 protein tends to localize in the cytoplasm, while acetylation at lysine 47 induces nuclear localization [41]. Interestingly, hypoxic stress induces the cytoplasm-to-nucleus translocation of eIF5A2 protein in esophageal squamous cell carcinoma (ESCC) cell lines, and eIF5A2 in the nucleus, promotes the transcription of HIF1α by binding to the promoter region of HIF1α [26].

#### Turnover of eIF5As

By measuring the hypusine pre-labelled by radioactive spermidine after a chase with unlabelled spermidine, the half-life of total eIF5A1 protein (hypusine-containing protein) was measured to be longer than 24 h in rat hepatoma cells [59]. In human cells, eIF5A1 was ubiquitinated by E3 ubiquitin-protein ligase CHIP, the degradation of this protein was via proteasome pathway, and the half-life of eIF5A1 was 29.1 h. [60]. Furthermore, it can be observed that eIF5A1 negatively correlates with E3 ubiquitin-protein ligase CHIP in colorectal tumor tissues. The stability also can be affected by exogenous stimuli. It was revealed that colon cancer cell lines were sensitive to acute heat stress, which can be explained by the induced loss of eIF5A1 protein [61]. Further results show that the loss of eIF5A1 protein was owing to enhanced protein degradation, and half-life of eIF5A1 was less than 30 min after acute heat stress treatment while it was demonstrated to be longer than 20 h in control cells. For the sake of simplicity, we summarized and presented the information of this section in Table 1.

**Table 1:** Post-translational modifications of human eIF5A1 and eIF5A2

Protein	Hypusination	Acetylation	Acetyl-hypusination	Sulfation	Ubiquitylation	References
eIF5A1	Lysine 50	Lysine 47	Lysine 50	Tyrosine 69	+	[1, 42, 44, 45, 48, 60]
eIF5A2	Lysine 50	Lysine 47	ND	ND	ND	[41]

+ site(s) unidentified, *ND* no data

## Functions of eIF5As in cancers

### eIF5A1 in cancers: tumor suppressor gene or oncogene?

#### Tumor suppressor gene

Apoptosis is a highly conserved process, which can be initiated by the intrinsic pathway or the extrinsic pathway, and both pathways of apoptosis are important to ensure fine-tuning of life cycle of multicellular organisms and the elimination of defective cells [62]. Thus, its dysregulation can cause many diseases. From a cancer perspective, the induction of apoptosis in precancerous lesions can remove potentially harmful cells, and unchecked cell proliferation often triggers apoptosis; thereby, evasion from apoptosis is considered a fundamental hallmark of carcinogenesis [63]. Several pieces of evidence demonstrated that eIF5A1 is a critical apoptosis regulator (reviewed in [64]). In lymphoma, eIF5A1 has been identified as a tumor suppressor via promoting apoptosis [65]. Short hairpin RNA against *EIF5A1* promoted lymphomagenesis in a mouse lymphoma model, and led to the reduction of apoptosis in pre-malignant B cells. In contrast, apoptosis can be induced by enforced expression of a wild-type (but not mutant) eIF5A in eIF5A-deleted cell lines. Additionally, low eIF5A protein expression is associated with a worse progression-free survival in patients with diffuse large B cell lymphomas. Furthermore, results suggested that knockdown of eIF5A1 led to the reduction of apoptosis through the down-regulation of the apoptosis regulator BAX protein, although how *BAX* is controlled by eIF5A1 remains unclear.

As an essential self-digesting process that is involved in recycling of intracellular material and maintaining cellular homeostasis, autophagy may be the key in maintaining human health, and its dysregulation is correlated with multiple human diseases [66]. Recent reports demonstrate that eIF5A1 can promote autophagy by regulating the translation of autophagy-related proteins. By high-throughput RNAi screen, eIF5A1 was identified as a critical autophagy regulator, and further results confirmed that eIF5A1 depletion disrupts autophagosome formation via decreasing the efficiency of the translation of *ATG3* mRNA [10]. Additionally, hypusinated eIF5A1 acts as a positive regulator of autophagy by promoting the translation of triproline motif of the transcription factor EB, which is essential for the expression of genes involved in autophagy [67]. Although autophagy may be needed for cancer cell survival, it directly and indirectly suppresses tumor initiation. The role of eIF5A1-mediate autophagy in cancers development would need to be further studied.

#### Oncogene

As early as 1984, the involvement of hypusine in cell proliferation has been documented [68]. Up to now, it was reported that eIF5A1 protein and hypusination pathway play an important role in the progression of cancer. In glioblastomas, the relevant investigation reported an up-regulation of *DHS*, *DOHH*, and *EIF5A1* in glioblastoma patient samples with different grades, and both inhibition of hypusination by GC7 or stable knockdown of *DHS* and *EIF5A1* impaired the proliferation of glioma cells in vitro [69]. In colorectal cancer (CRC), *EIF5A1* was demonstrated to be upregulated compared with normal colorectal tissues using mRNA PCR arrays, and its high expression is an indicator of poor prognosis [70]. In cervical tumors, immunohistochemical studies showed a robust eIF5A1 protein expression, and siRNAs directed against *EIF5A1* or *DOHH* showed an antiproliferative effects in HeLa cells [71]. In epithelial ovarian cancer (EOC), both western blotting and IHC staining assay revealed that eIF5A1 protein expression was upregulated compared with the normal ovarian tissues, and the results of correlation regression analysis and Kaplan–Meier analysis indicated that higher eIF5A1 protein levels are correlated with EOC progression and poor prognosis. Further studies of ectopic eIF5A1 expression showed that enhanced eIF5A1 expression can promote cell proliferative, migration, and invasive capabilities of EOC cell lines and also induced epithelial-mesenchymal transition (EMT) [72].

The molecular mechanisms whereby eIF5A1 promote tumor progression remain unclear, though advances have been made in studies involved in pancreatic cancer. About 90% of pancreatic cancer is pancreatic ductal adenocarcinoma (PDAC) originated from pancreatic ductal epithelium with an overall survival rate of less than 5%. In recent years, works by Klemke’s group demonstrated that eIF5A1 was involved in PDAC pathogenesis and metastasis, and suggested that eIF5A1 protein was a promise therapeutic target. Pseudopodium-enriched atypical kinase 1 (PEAK1) is a newly identified non-receptor tyrosine kinase, which plays an essential role in driving PDAC malignancy, but the downstream signaling processes that mediate its function are uncharacterized. They found that KRas/eIF5A1/PEAK1 as a new signaling axis which contributes to PDAC growth in vitro and in vivo [21], and through a proteomics analysis of PEAK1-depleted PDAC cells, they demonstrated that Yes-associated protein 1 which is a central cytoskeletal sensor and transcriptional coactivator functions as a downstream target of eIF5A1-PEAK1 signaling [73]. Interestingly, they proved that eIF5A1-PEAK1 signaling modulate the *KRAS* gene expression in turn [74]. Lastly, they also demonstrated that eIF5A1 plays a role in pancreatic cancer metastasis via regulating *RhoA* and *ROCK2* gene expression post-transcriptionally, both of which are mainly associated with cytoskeleton organization [75].

### eIF5A2 in cancer development and progression

#### Bladder carcinoma

Urothelial carcinoma of the bladder is the most common histopathologic type of bladder cancer (BC), and the expression of eIF5A2 protein serves as a useful molecular marker to predict outcome in patients with urothelial carcinoma of the bladder, after patients were treated with radical cystectomy [76]. In pTa/pT1 urothelial carcinoma of the bladder, patients with high eIF5A2 protein levels in carcinoma tissues had shorter mean recurrence-free survival time and mean progression-free survival time than that of patients with low expression in carcinoma tissues [77]. In another study, western blot results showed that the expression of eIF5A2 was upregulated in 10 fresh BC tissues as compared with their adjacent normal bladder tissues, and among the 154 patients with localised invasive BC treated with radical cystectomy, high eIF5A2 expression in cancer tissues had significantly poor metastasis-free survival. What is more, overexpression of *EIF5A2* promoted cell’s motility both in vitro and in vivo, and induced EMT in BC cells, but knockdown of *EIF5A2* has opposite effect. Further studies revealed that eIF5A2 promotes BC cell aggressiveness by up-regulation of TGF-β1, a key driver of EMT [78]. Thus, eIF5A2 serves as a promising therapeutic target for treatment of BC. SiRNA against *EIF5A2* can be efficiently delivered to the tumor sites by Mg(II)-catechin nanoparticles via tail vein injection in vivo, and inhibited tumor growth both in subcutaneous xenograft model and in situ BC model [79].

#### Cervical carcinoma

In cervical carcinoma, both *EIF5A2* mRNA and protein levels are upregulated in cervical cancer tissues as compared with those in adjacent non-tumor tissues. Higher *EIF5A2* expression was correlated with aggressive characteristics of cervical cancer, and serves as a biomarker for shorter overall survival and disease-free survival in FIGO stage II patients and in patients with a negative pelvic lymph node status [80]. Knockdown of *EIF5A2* inhibited in vivo tumorigenic ability, attenuated cell growth, induced cell cycle arrest and decreased the cell migration ability of HeLa cells. Additionally, the authors revealed that eIF5A2 exerts its effect on viability and mobility of HeLa cells by an RhoA/ROCK-dependent manner [81].

#### Colorectal cancer

In CRC, higher *EIF5A2* expression was correlated with aggressive characteristics of cancers and poor survival of patients [82, 83]. Knockdown of *EIF5A2* prevented CRC cell invasiveness and inhibited EMT, but overexpression of *EIF5A2* enhanced cell invasiveness and induced EMT. Furthermore, the metastasis-associated protein 1 is required for eIF5A2-induced CRC cell invasiveness and EMT [83].

#### Esophageal squamous cell carcinoma

In ESCC, increased *EIF5A2* mRNA was observed in tumor tissues compared with paired non-tumor tissues, and according to immunohistochemistry data of 232 tumor tissues and 215 non-tumor tissues, tumor tissues showed higher frequency of positive staining of eIF5A2 compared with non-tumor tissues [26]. The authors also disclosed that eIF5A2 contributes to metastasis and angiogenesis in ESCC, and is a promising target for treatment.

#### Gastric cancer

In gastric cancer (GC), the overexpression of *EIF5A2* was correlated with worse clinicopathological features moreover, patients with higher *EIF5A2* expression ad shorter mean survival time [84]. Knockdown of *EIF5A2* caused an apparent suppression of proliferation, migration, and invasion in GC cells. In contrast, enforced expression of *EIF5A2* results in an opposite effect [85].

#### Hepatocellular carcinoma

In hepatocellular carcinoma (HCC), mRNA level of *EIF5A2* was upregulated in more than half of HCC clinical samples. Kaplan–Meier analysis showed that *EIF5A2* overexpression was significantly associated with shorter survival time of patients [86]. Ectopic expression of *EIF5A2* in HCC cell lines significantly promoted cell growth and cell motility in vitro and in vivo, but siRNAs targeting *EIF5A2* inhibited proliferation and cell motility [87]. The cellular processes which can be regulated by eIF5A2 in HCC including maintenance of cancer stem cells [88], metabolic reprogramming [86], EMT [87], and reorganization of actin cytoskeleton [87].

#### Lung cancer

According to semiquantitative scoring criterion for IHC of eIF5A2, in which both staining intensity and positive areas were recorded, the expression of eIF5A2 in all normal lung tissue was absent or at low levels with staining index less than or equal to 3, but higher protein levels of eIF5A2 was observed in many of non-small cell lung cancer (NSCLC) specimens with staining index higher than 3, and higher eIF5A2 protein expression predicts poor survival for stage I NSCLC patients [89]. By western blot assay, increased eIF5A2 expression was observed in NSCLC cell lines (A549, H23, Calu-3, H1299, and H460 cells) as compared with the benign human bronchial epithelial HBE cell line, and *EIF5A2* silencing can inhibit the cell’s motility and growth ability of H1299 and H460 cells [90]. In A549 cells, E-cadherin expression was increased and vimentin expression decreased significantly after down-regulation of *EIF5A2* expression by siRNA, suggesting eIF5A2 might play an important role in promoting A549 NSCLC cells to undergo EMT [91].

#### Melanoma

In human melanoma, both the cytoplasmic and nuclear eIF5A2 protein levels are inversely correlated with patient survival, and patients with positive cytoplasmic and nuclear eIF5A2 staining showed the poorest disease-specific 5-year survival rates compared with patients with negative staining in both the cytoplasm and nucleus and patients with only positive stain in either the cytoplasm or nucleus [92, 93]. Though enforced expression of *EIF5A2* and *EIF5A2* knockdown has no effect on apoptosis or cell proliferation in melanoma cell lines, cell invasion was significantly increased and decreased, respectively. Also, eIF5A2 may induce EMT as demonstrated by increased mesenchymal markers and decreased epithelial marker in melanoma cell line with *EIF5A2* overexpression. Furthermore, knockdown of matrix metalloproteinase-2 (MMP2) which was shown to have a crucial role in cell invasion diminished the effect of *EIF5A2* overexpression on cell invasion, suggesting MMP2, at least in part, mediates eIF5A2-induced invasion [93].

#### Ovarian carcinoma

According to eIF5A2 protein level in ovarian carcinoma tissues, patents with higher eIF5A2 protein levels had aggressive clinicopathologic features and poor survival [94]. In UACC-1598 cells, an ovarian cancer cell line, the cell growth rate was inhibited by antisense DNA sequence against *EIF5A2* [95].

#### Pancreatic cancer

Comparing the relative expression levels of eIF5A2 protein between normal and PDAC tissues revealed that eIF5A2 protein levels are amplified in human PDAC [21]. Furthermore, knockdown of eIF5A2 proteins in PDAC cells (PANC1 and 779E cells) inhibited their growth in vitro, whereas amplification of eIF5A2 proteins increased PDAC cell growth. In vivo, *EIF5A2* depletion in the 779E cells reduced tumor growth, while *EIF5A2* overexpression in PANC1 cells enhanced tumor growth. The downstream gene which is essential for eIF5A2 function is demonstrated to be PEAK1 non-receptor tyrosine kinase. Another paper revealed that the Shh-Gli1 signaling pathway, a key pathway in controlling cell growth and differentiation, regulates the transcription of *EIF5A2* gene in pancreatic cancer cells [27].

#### Other cancers

Expression of *EIF5A2* also predicts poor prognosis of nasopharyngeal carcinoma patients [96], prostate cancer patients [97], and upper tract urothelial carcinoma patients [98], however the exact functions of eIF5A2 in these cancers should be further studied.

### Roles of eIF5A1 in other diseases

#### Viral infections

Several eukaryotic translation initiation factors were suggested as a host cell cofactor for viruses replication and propagation, including eIF5A1. First, the viral trans-activator protein Rev, which is required for the expression of structural proteins of HIV-1, was demonstrated to specifically interact with eIF5A1 in HeLa cells, and the transactivation function of Rev was mediated by this specific interaction [51]. Second, eIF5A1 is required for the function of the viral trans-activator protein Rex which is necessary for the expression of structural proteins of human T-cell leukemia virus type I [99]. Additionally, eIF5A1 is involved in both internal ribosome entry site (IRES)-mediated HIV-1 RNA translation and cap-dependent RNA translation [100], and inhibition of hypusine formation impairs the IRES-mediated translation initiation at several retroviral IRESs [101, 102]. For Ebola virus (EBOV), blocking of hypusine formation inhibited EBOV gene expression and decreased the expression level of hexameric zinc-finger protein VP30, an essential component of the viral polymerase [103].

#### Diabetes

Diabetes mellitus characterized by high blood glucose levels is a condition caused by the dysfunction and/or destruction of insulin-producing pancreatic islet β cells. There are two forms of diabetes: type 1 diabetes mellitus, in which islet β cells are destroyed by an autoimmune response and type 2 diabetes mellitus which results from a progressive defect in insulin secretion in individuals with insulin resistance [104]. In both type 1 and type 2 diabetes, the early pathogenesis of β cell dysfunction results in part from the local release of pro-inflammatory cytokines; therefore, the understanding of molecular pathways by which pancreatic islet β cells response to pro-inflammatory cytokines could greatly contribute to the development of therapies. In inflammatory mouse models of diabetes, it was reported that both depletion of *EIF5A1* and inhibition of hypusination can improve glucose intolerance, and in vitro, depletion of *EIF5A1* can attenuate the pro-inflammatory cytokine-induced dysfunction of islets. These results suggested that eIF5A1 is required for islet β cells dysfunction induced by pro-inflammatory cytokines [105]. Furthermore, the authors revealed that the hypusinated eIF5A1 is essential for the synthesis of inducible nitric oxide synthase which mediates the cytokine-induced islet death by promoting the production of nitric oxide.

The pathogenesis of type 1 diabetes mellitus involves the innate and adaptive immune responses against various β-cell antigen, resulting in infiltration of pancreatic islets by cells of the immune system [106]. In the humanized mouse model of type 1 diabetes, there are evidences that inhibition of hypusination by GC7 reduces T helper 1 and T helper 17 cells which have a role in β-cell death, but enriches T-regulatory cells which can prevent undesirable immune responses in the pancreatic microenvironment [107]. In a non-obese diabetic mouse model, a reduction in diabetogenic T helper 1 cells was also observed in the pancreatic lymph nodes after injections of GC7 [108]. These results suggest that hypusinated eIF5A also can affect the immune cells response during type 1 diabetes mellitus pathogenesis.

#### Neural diseases

Spinal cord transaction (SCT), a serious damage to nerves which run inside the vertebral column, results in irreversible loss of function at the level and below of injured location, and currently there is no cure [109]. A major reason for the poor prognosis of SCT is associated with neuronal loss and the extremely weak regenerative capacity of axons of the central nervous system after injury; although there is some inherent regenerative capacity of the central nervous system, it is very limited [110]. A recent study revealed the association of eIF5A1 with the neuroplasticity and functional recovery after SCT [111]. In SCT rats, overexpression of *EIF5A1* increased motor function recovery after SCT compared to control group while *EIF5A1* knockdown had an opposite result. In vitro, overexpressing *EIF5A1* in primary neurons significantly increased cell number and neurite length as compared with the control group whereas a decrease in the number of neurons and neurite length was observed in primary neurons with *EIF5A1* knockdown. Furthermore, Rho GDP dissociation inhibitor alpha (RhoGDIα) which was identified as a negative regulator of RHO GTPases [112], is required for the function of eIF5A1. The effect of eIF5A1 down-regulation was inhibited when RhoGDIα was upregulated, and the effect of eIF5A1 down-regulation was rescued by RhoGDIα up-regulation.

Cerebral ischemia/reperfusion injury can cause neurological deficit, loss of cognitive function, and severe brain damage [113]. A recent report demonstrated that tyrosine-sulfated form of eIF5A1 (termed as oxidative stress-responsive apoptosis inducing protein [ORAIP]) mediated apoptosis of cerebral neurons in ischemia/reperfusion injury [114]. The authors found that ORAIP expression was increased in cerebral cells of rats subjected to cerebral ischemia (60 min) and reperfusion (15 min), and the ORAIP concentration in the cerebrospinal fluid was also increased. More importantly, both pre-treatment and post-treatment with anti-ORAIP mAb which neutralize ORAIP can suppress cerebral ischemia/reperfusion injury in vivo, suggesting ORAIP is a promising therapeutic target for treatment of cerebral ischemia/reperfusion injury.

## Conclusions and future perspectives in eIF5As research

In this review, we summarize the detailed knowledge about *EIF5A1* and *EIF5A2* in a variety of human diseases, especially in human cancers. *EIF5A1* was identified as an oncogene in glioblastoma [69], CRC [70], cervical tumors [71], ovarian cancer [72], and PDAC [21], and can be served as a prognostic factor for poor survival in CRC and EOC patients [70, 72]. Moreover, *EIF5A1* does not only act as an oncogene but also functions as a tumor suppressor. It has been demonstrated that *EIF5A1* functions as a tumor suppressor in lymphoma via promoting apoptosis, and low eIF5A protein expression is associated with a worse progression-free survival in patients with diffuse large B-cell lymphomas [65]. For *EIF5A2*, it acts as an oncogene in several cancers. Its high expression correlates with poor survival and aggressive tumor biology, including BC [76–78], cervical cancer [80], CRC [82, 83], ESCC [26], GC [84], HCC [86], NSCLC [89], melanoma [92, 93], ovarian carcinoma [94], nasopharyngeal carcinoma [96], prostate cancer [97], and upper tract urothelial carcinoma [98]. Altogether, both *EIF5A1* and *EIF5A2* are involved in the cancer development and progression, and can be a useful marker for diagnosis and prognosis.

eIF5A1 and eF5A2 exert their role in promoting cancer cell’s growth, invasion, and metastasis ability via a variety of cellular processes including maintenance of cancer stem cells [88], metabolic reprogramming [86], EMT [72, 78, 83, 87, 91, 93], and reorganization of actin [87], therefore silencing *EIF5A1* or *EIF5A2* expression is a potential strategy for anticancer therapy, such as for ESCC [26], BC [79], and multiple myeloma [115]. Moreover, GC7 treatment which inhibited the formation of hypusine can enhance the chemosensitivity of cancer cells, such as HCC cells [116], estrogen receptor negative breast cancer cells [117], PDAC cells [118], BC cells [119], oral cancer cells [120, 121], and acute lymphoblastic leukemia cells [122]. The exact molecular mechanisms of these two proteins in cancer development and progression still remain unclear. One potential mechanism may be implicated in translational control of specific mRNAs, since more and more reports disclosed that eIF5A1 can promote the translation elongation of mRNA with ribosome stalling motifs [10, 67, 123], and it was also revealed that eIF5A1 can promote the translation of mRNA by promoting its nuclear export [105]. Another potential mechanism is as transcription factor in the nucleus, as eIF5A2 was observed to bind to the promoter region of HIF1α and regulate HIF1α transcription in ESCC cell lines [26].

Given the fact that eIF5A1 and eIF5A2 are shuttling proteins and present in both the nucleus and cytoplasm, however, very little is known about their functions in the nucleus and the residence of eIF5A1 isoform 2 in the mitochondrion; in which all these are interesting areas to be further studied for eIF5As research in the future.

## Data Availability

All data generated or analyzed during this study are included in this published article.

## Abbreviations

BC: Bladder cancer
CRC: Colorectal cancer
DHS: Deoxyhypusine synthase
DOHH: Deoxyhypusine hydroxylase
EBOV: Ebola virus
EIF5A: Eukaryotic translation initiation factor 5A
EMT: Epithelial–mesenchymal transition
EOC: Epithelial ovarian cancer
ESCC: Esophageal squamous cell carcinoma
GC: Gastric cancer
GC7: N1-guanyl-1,7-diaminoheptane
Gli1: GLI family zinc finger protein 1
HCC: Hepatocellular carcinoma
HIF1α: Hypoxia-inducible factor 1α
HIV-1: Human immunodeficiency virus type 1
IRES: Internal ribosome entry site
MMP2: Matrix metalloproteinase-2
NSCLC: Non-small cell lung cancer
ORAIP: Oxidative stress-responsive apoptosis inducing protein
PDAC: Pancreatic ductal adenocarcinoma
PEAK1: Pseudopodium-enriched atypical kinase 1
PTM: Post-translational modification
RhoGDIα: Rho GDP dissociation inhibitor alpha
SCT: Spinal cord transaction
Shh: Sonic hedgehog
